# Awareness of HPV-associated oropharyngeal cancers among GPs in The Netherlands: a cross-sectional study

**DOI:** 10.3399/BJGPO.2021.0080

**Published:** 2021-12-15

**Authors:** Imke Demers, Femke Verhees, Leo J Schouten, Jean WM Muris, Bernd Kremer, Ernst Jan M Speel

**Affiliations:** 1 Department of Pathology, GROW-school for Oncology and Developmental Biology, Maastricht University Medical Center, Maastricht, The Netherlands; 2 Department of Otorhinolaryngology, Head and Neck Surgery, GROW-school for Oncology and Developmental Biology, Maastricht University Medical Center, Maastricht, The Netherlands; 3 Department of Epidemiology, GROW-school for Oncology and Developmental Biology, Maastricht University Medical Center, Maastricht, The Netherlands; 4 Department of Family Medicine, CAPHRI Care and Public Health Research Institute, Maastricht University Medical Center, Maastricht, The Netherlands

**Keywords:** cross-sectional studies, general practitioners, human papillomavirus 16, oropharyngeal neoplasms, primary health care, risk factors

## Abstract

**Background:**

The incidence of human papillomavirus (HPV)-associated oropharyngeal cancer (OPC) is increasing in high income countries. HPV-associated OPC generally presents as an invasive disease, often with lymph node involvement, in relatively young patients with minimal or no history of smoking and alcohol consumption. Knowledge on HPV-associated OPC among primary care professionals is essential for disease recognition and early start of treatment.

**Aim:**

To examine the knowledge on HPV-associated OPC among GPs in the Netherlands.

**Design & setting:**

A cross-sectional postal survey among GPs in the Netherlands.

**Method:**

A 12-item questionnaire was sent to 900 randomly selected general practices. Outcome measures included awareness of the link between HPV and OPC, epidemiological trends, and patient characteristics. Data were statistically analysed for sex, years after graduation, and self-rated knowledge of OPC.

**Results:**

A total of 207 GPs participated in this study. Seventy-two per cent recognised HPV as a risk factor for OPC and 76.3% were aware of the increasing incidence rate of HPV-associated OPC. In contrast, 35.7% of participants knew that patients with HPV-associated OPC are more often male, and just over half (53.6%) of the participants were aware of the younger age of these patients.

**Conclusion:**

More than one-quarter of GPs in the Netherlands are unaware of HPV as a causative factor for OPC. Furthermore, there is a gap in knowledge on characteristics of patients with HPV-associated OPC . Further training on these topics could improve disease recognition and, ultimately, patient survival.

## How this fits in

Since HPV-associated OPC generally presents in a group of relatively young patients without typical risk factors, disease recognition can pose challenges for GPs without detailed knowledge of the disease and corresponding patient characteristics. A meta-analysis on the knowledge on HPV-associated OPC among different populations revealed that the knowledge on HPV in OPC among medical and dental professionals varied from 26–91%. In the current study, the awareness of the link between HPV and OPC, including epidemiological trends and demographic patient profiles, among GPs in the Netherlands was investigated for the first time. The results of this study identify areas where further education for GPs is needed to increase specific knowledge to improve disease recognition and patient outcomes.

## Introduction

Head and neck cancer (HNC) was the seventh most common cancer worldwide in 2018, accounting for 3% of all cancers.^1^ Five-year, age-standardised relative survival rates range from 25–60%, depending on anatomical location, HPV status, and stage at diagnosis.^2^ HNC is usually diagnosed in older patients in association with tobacco use and heavy alcohol consumption.^3–5^ In addition, infection with high-risk HPV, primarily HPV type 16, has been recognised as a major risk factor for the development of HNC, specifically OPC. Partly as a result of the worldwide decline in tobacco use, the incidence of HNC has decreased over recent decades. Conversely, the incidence of HPV-associated OPC is increasing in so-called ‘high income’ countries, including Australia, the US, Canada, Sweden, Denmark, and the Netherlands.^3,6–9^ A meta-analysis including 5396 OPCs observed an increase in the proportion of HPV-related OPC from 40.5% before 2000 to 72.2% after 2005, with significant increases in North America and Europe.^10^ In the Netherlands, an increase in the prevalence of HPV in OPC was observed from 5.1% in 1990 to 29% in 2010.^9^ More recent studies showed a prevalence of HPV in 30–50% of the OPC cases in the Netherlands.^11–13^

HPV-associated OPC is considered to be a distinct clinical and molecular entity.^14,15^ In contrast to patients with non-HPV-associated OPC, patients with HPV-associated OPC are younger, more often male, have a higher socioeconomic status and more lifelong sexual partners, and are less likely to have a history of extensive tobacco and alcohol use.^3,15,16^ Compared with non-HPV-associated tumours, HPV-associated tumours are generally characterised by a better prognosis, primarily because they are more responsive to chemotherapy and radiotherapy.^17,18^ Despite this beneficial treatment response, HPV-associated tumours often have a peculiar clinical presentation. Compared with non-HPV-associated tumours, HPV-associated tumours generally present as smaller (asymptomatic) tumours, but often with regional lymph node metastases and sometimes even with presentation of neck metastases from an occult primary tumour.^19–21^ Diagnosis of oropharyngeal HPV-associated tumours at earlier disease stage is associated with improved overall and disease-specific survival rates.^22^ Furthermore, HPV-associated OPC precursor lesions are scarce, unlike cervical cancer, which means that no validated preventive screening method has been developed for these tumours.^23–25^ Therefore, early disease recognition by primary care professionals and no delay in treatment are crucial for patient outcomes.

Recognising patients at risk of HPV-associated OPC can pose challenges for GPs, who may not have detailed knowledge of the disease and corresponding patient characteristics. A systematic review by Dodd *et al* identified 41 studies investigating the knowledge about the link between HPV and OPC in different populations.^26^ This study revealed that the lowest knowledge was observed in the general population (1–44%), which can be confirmed in a recent study in the Netherlands showing that only 11% of the general population was aware of the link between HPV and OPC (29.2% of people stated they were aware of the existence of HPV).^27^ The same systematic review reported that the highest knowledge on HPV in OPC was reported among medical and dental professionals (26–91%), which was also found by a recent study by Lechner *et al* in the UK, reporting that 74% of GPs recognised HPV as a risk factor for OPC.^28^

This study is the first to assess awareness of the link between HPV and OPC, the epidemiological trends in (HPV-associated) OPC and demographic profiles of patients with HPV-associated OPC among a randomly selected group of GPs in the Netherlands. The results might identify areas where further education for GPs are needed to increase specific knowledge, and thereby improve disease recognition and patient outcomes.

## Method

### Survey design

A cross-sectional questionnaire survey was performed among GPs in the Netherlands. A short questionnaire was adapted and translated from an already developed questionnaire by Lechner *et al* ^28^ (Supplementary File S1). This questionnaire assessed demographic characteristics of participants, self-rated knowledge of OPC, awareness of OPC risk factors, knowledge on the association between HPV and OPC, and characteristics of patients with HPV-associated OPC. Demographic characteristics included sex, years since graduation, and current position. Self-rated knowledge on OPC was assessed by a Likert scale. To assess the awareness of risk factors, 11 risk factors (of which eight were correct and three were false) were selected from epidemiological literature.

### Participants

The postal addresses of 900 GPs throughout the Netherlands were obtained from the Netherlands Institute for Health Services Research (NIVEL). These 900 GPs were selected by random sampling of all GPs registered at NIVEL, comprising approximately 85–90% of all GPs in the Netherlands. A response rate of 20% was anticipated based on previous surveys among GPs (NIVEL, institutional communication). The questionnaire was administered in September 2020 to the GPs by mail. To increase the response rate, questionnaires could be completed both in paper format and by a link to the online platform Survey Monkey. In addition, a reminder was sent 2 weeks after the initial invitation. Answers of returned paper questionnaires were added as separate collectors to the Survey Monkey database. Both paper format and online questionnaires were collected anonymously. After completing the questionnaire, participants were given a factsheet with information about HPV and HPV-associated OPC (Supplementary file 2).

### Statistical analysis

Statistical analyses were performed using SPSS statistical software for Windows (version 20), and Stata (version 14.1). Descriptive analyses with calculated measures of central tendency and variation were computed, along with frequency tables for categorical variables. Whether distributions of categories are different was tested using χ^2^ tests and likelihood ratio tests. The extended Mantel–Haenszel stratified test of association was used to test for linear trends. For this, variables were recoded into two categories (the ‘correct’ answers and ‘incorrect answers’). *P* values below 0.05 were considered statistically significant.

## Results

### Participants' characteristics

The questionnaire was sent to 900 GPs throughout the Netherlands. Overall, 212 questionnaires were collected, resulting in a response rate of 23.6%. The majority of the questionnaires were completed in paper format compared with the online questionnaire (141 versus 71). Five questionnaires were incomplete (6–9 missing answers of 12 questions in total) and therefore excluded from analysis. The demographic characteristics of participants are shown in Table 1. Owing to the applied privacy legislation, it was not possible to compare features between responders and non-responders. Nevertheless, responders could be compared with the whole registry of GPs in the Netherlands (in 2019) for sex, current position, and GP experience.^29,30^ Supplementary Table S1 shows that only the percentage of female GPs is different in the whole registry (58%) versus the present study population (48%). Notably, 49 out of 207 responding GPs (23.7%) rated their knowledge of OPC as ‘poor’.

**Table 1.** Demographic characteristics and self-rated knowledge of OPC of 207 participating GPs in the Netherlands (2020)

Characteristics	*n*	%
Stage of training or position		
GPST year 1	2	1
GPST year 2	0	0
GPST year 3	7	3.4
GP	198	95.7
Sex		
Male	107	51.7
Female	100	48.3
Years since graduation		
Still in training	9	4.3
<2 years	7	3.4
2–5 years	18	8.7
5–10 years	39	18.8
10–20 years	59	28.5
>20 years	75	36.2
Self-rated knowledge of OPC		
Poor	49	23.7
Sufficient	148	71.5
Good	10	4,8
Very good	0	0

GPST = general practitioner specialty training. OPC = oropharyngeal cancer.

### Knowledge of HPV and risk factors for OPC

Of all 207 responders, 72.0% were aware of the link between HPV infection and OPC; 23.7% were not aware of this link and 4.3% were not sure (Table 2). To assess awareness of risk factors for OPC in general, responders were confronted with 11 risk factors and asked whether these present risk factors for OPC or not (Table 3). Infection with HPV was recognised as a risk factor for OPC by 78.7% of participants. Participants had good knowledge of the risk factors smoking, alcohol abuse, and chewing of tobacco (100%, 98.1%, and 91.3%, respectively). Chewing of betel leaf, betel palm, or betel nut (Areca nut); poor oral hygiene; family history; and low fruit and vegetable consumption were less well recognised as risk factors (28.0%, 51.7%, 56.5%, and 31.4%, respectively).

**Table 2.** Knowledge of HPV as risk factor for OPC and epidemiological trends of OPC incidence among 207 GPs in the Netherlands (2020)

		Total, *n* (%)	Sex, *n* (%)			Years after graduation as GP, *n* (%)						Self-rated knowledge of OPC, *n* (%)			
			Female	Male	*P* value	<2^a^	2–5	5–10	10–20	>20	*P* value	Poor	Sufficient	Good	*P* value
Were you aware of the link between HPV and OPC before today?	Yes	149 (72.0%)	80 (74.8%)	69 (69.0%)	0.273	14 (87.5%)	14 (77.8%)	31 (79.5%)	39 (66.1%)	51 (68.0%)	0.267	29 (59.2%)	112 (75.7%)	8 (80.0%)	0.216
	No	49 (23.7%)	21 (19.6%)	28 (28.0%)		2 (12.5%)	2 (11.1%)	7 (17.9%)	16 (27.1%)	22 (29.3)		17 (34.7%)	30 (20.3%)	2 (20.0%)	
	Not sure	9 (4.3%)	6 (5.6%)	3 (3.0%)		0 (0.0%)	2 (11.1%)	1 (2.6%)	4 (6.8%)	2 (2.7%)		3 (6.1%)	6 (4.1%)	0 (0.0%)	
	Total	207 (100%)	107 (100%)	100 (100%)		16 (100%)	18 (100%)	39 (100%)	59 (100%)	75 (100%)		49 (100%)	148 (100%)	10 (100%)	
														
Over the past two decades, HPV- associated OPC rates have:	Increased	158 (76.3%)	80 (74.8%)	78 (78.0%)	0.135	10 (62.5%)	11 (61.1%)	35 (89.7%)	42 (71.2%)	60 (80.0%)	0.020^b^	36 (73.5%)	114 (77.0%)	8 (80.0%)	0.664
	Decreased	6 (2.9%)	2 (1.9%)	4 (4.0%)		2 (12.5%)	2 (11.1%)	0 (0.0%)	0 (0.0%)	2 (2.7%)		1 (2.0%)	5 (3.4%)	0 (0.0%)	
	Stayed the same	8 (3.9%)	7 (6.5%)	1 (1.0%)		2 (12.5%)	1 (5.6%)	2 (5.1%)	2 (3.4%)	1 (1.3%)		4 (8.2%)	4 (2.7%)	0 (0.0%)	
	Not sure	35 (16.9%)	18 (16.8%)	17 (17.0%)		2 (12.5%)	4 (22.2%)	2 (5.1%)	15 (25.4%)	12 (16.0%)		8 (16.3%)	25 (16.9%)	2 (20.0%)	
	Total	207 (100%)	107 (100%)	100 (100%)		16 (100%)	18 (100%)	39 (100%)	59 (100%)	75 (100%)		49 (100%)	148 (100%)	10 (100%)	
Over the past two decades. smoking- associated OPC rates have:	Increased	96 (46.4%)	58 (54.2%)	38 (38.0%)	0.021	7 (43.8%)	10 (55.6%)	19 (48.7%)	26 (44.1%)	34 (45.3%)	0.354	26 (53.1%)	64 (43.2%)	6 (60.0%)	0.219
	Decreased	41 (19.8%)	15 (14.0%)	26 (26.0%)		4 (25.0%)	4 (22.2%)	8 (20.5%)	13 (22.0%)	12 (16.0%)		5 (10.2%)	34 (23.0%)	2 (20.0%)	
	Stayed the same	42 (20.3%)	17 (15.9%)	25 (25.0%)		4 (25.0%)	4 (22.2%)	4 (10.3%)	10 (16.9%)	20 (26.7%)		9 (18.4%)	31 (20.9%)	2 (20.0%)	
	Not sure	28 (13.5%)	17 (15.9%)	11 (11.0%)		1 (6.3%)	0 (0.0%)	8 (20.5%)	10 (16.9%)	9 (12.0%)		9 (18.4%)	19 (12.8%)	0 (0.0%)	
	Total	207 (100%)	107 (100%)	100 (100%)		16 (100%)	18 (100%)	39 (100%)	59 (100%)	75 (100%)		49 (100%)	148 (100%)	10 (100%)	

^a^Also includes GPs still in training. ^b^No statistically significant trend observed with the extended Mantel-Haenszel test. *P* values were calculated with χ^2^ tests or likelihood ratio tests. HPV = human papillomavirus. OPC = oropharyngeal cancer.

**Table 3.** Knowledge of reported risk factors for oropharyngeal cancer among 207 GPs in the Netherlands (2020)

Risk factor	Yes		No		Not sure	
	*n*	%	*n*	%	*n*	%
Smoking	207	100.0	0	0.0	0	0.0
Alcohol abuse	203	98.1	1	0.5	3	1.4
Chewing of tobacco	189	91.3	4	1.9	14	6.8
Chewing of betel leaf/palm/nut	58	28.0	12	5.8	137	66.2
Marijuana use	106	51.2	24	11.6	77	37.2
Poor oral hygiene	107	51.7	54	26.1	46	22.2
Herpes simplex virus infection	27	13.0	99	47.8	81	39.1
Human papillomavirus infection	163	78.7	9	4.3	35	16.9
Positive family history	117	56.5	40	19.3	50	24.2
Low fruit and vegetable consumption	65	31.4	47	22.7	95	45.9
Sun exposure	34	16.4	110	53.1	63	30.4

Herpes Simplex virus infection, marijuana use, and sun exposure are not proven risk factors for oropharyngeal cancer.

Over three-quarters of participants were aware of the increase of HPV-associated OPC cases over the past two decades (76.3%). A linear trend with years after graduation was not observed (*P* = 0.265). In contrast, only 19.8% were aware of the decrease in smoking-associated OPC rates during the same period. Interestingly, male GPs were significantly more aware of this decrease compared with female GPs (*P* = 0.021) (Table 2).

### Knowledge of HPV-associated OPC patient characteristics

Knowledge of HPV-associated OPC patient characteristics among GPs is essential for disease recognition and early start of treatment. Only 35.7% of all participants knew that OPC patients with HPV-associated tumours are more often male, and a comparable percentage (34.3%) did not know (Table 4). GPs who rated their knowledge of OPC as ‘good’ were more aware of this sex difference (*P* = 0.003). However, this is a small group of only 10 GPs (4.8% of total, Table 1) and a linear trend for self-rated knowledge of OPC and awareness of the male sex of patients was not observed (*P* = 0.152).

**Table 4.** Knowledge of HPV-associated OPC patient characteristics and prognosis among 207 GPs in the Netherlands (2020)

		Total, *n* (%)	Sex, *n* (%)			Years after graduation as GP, *n* (%)						Self-rated knowledge of OPC, *n* (%)			
			Female	Male	*P* value	<2^a^	2–5	5–10	10–20	>20	*P* value	Poor	Sufficient	Good	*P* value
OPC patients with HPV- associated tumours are more often:	Male	74 (35.7%)	38 (35.5%)	36 (36.0%)	0.415	6 (37.5%)	4 (22.2%)	17 (43.6%)	21 (35.6%)	26 (34.7%)	0.424	16 (32.7%)	51 (34.5%)	7 (70.0%)	0.003^b^
	Female	35 (16.9%)	14 (13.1)	21 (21.0%)		4 (25.0%)	4 (22.2%)	5 (12.8%)	11 (18.6%)	11 (14.7%)		3 (6.1%)	31 (20.9%)	1 (10.0%)	
	Equal	27 (13.0%)	16 (15.0%)	11 (11.0%)		1 (6.3%)	1 (5.6%)	8 (20.5%)	10 (16.9%)	7 (9.3%)		4 (8.2%)	23 (15.5%)	0 (0.0%)	
	Don’t know	71 (34.3%)	39 (36.4)	32 (32.0%)		5 (31.3%)	9 (50.0%)	9 (23.1%)	17 (28.8%)	31 (41.3%)		26 (53.1%)	43 (29.1%)	2 (20.0%)	
	Total	207 (100%)	107 (100%)	100 (100%)		16 (100%)	18 (100%)	39 (100%)	59 (100%)	75 (100%)		49 (100%)	148 (100%)	10 (100%)	
OPC patients with HPV- associated tumours are more often:	Age <60 years	111 (53.6%)	54 (50.5%)	57 (57.0%)	0.325	9 (56.3%)	10 (55.6%)	24 (61.5%)	30 (50.8%)	38 (50.7%)	0.871	23 (46.9%)	86 (58.1%)	2 (20.0%)	0.018^b^
	Age >60 years	42 (20.3%)	26 (24.3%)	16 (16.0%)		4 (25.0%)	4 (22.2%)	8 (20.5%)	13 (22.0%)	13 (17.3%)		8 (16.3%)	28 (18.9%)	6 (60.0%)	
	Don’t know	54 (26.1%)	27 (25.2%)	27 (27.0%)		3 (18.8%)	4 (22.2%)	7 (17.9%)	16 (27.1%)	24 (32.0%)		18 (36.7%)	34 (23.0%)	2 (20.0%)	
	Total	207 (100%)	107 (100%)	100 (100%)		16 (100%)	18 (100%)	39 (100%)	59 (100%)	75 (100%)		49 (100%)	148 (100%)	10 (100%)	
The prognosis of patients with HPV-positive OPC is generally ... compared with HPV-negative OPC	Better	36 (17.4%)	18 (16.8%)	18 (18.0%)	0.292	6 (37.5%)	3 (16.7%)	6 (15.4%)	14 (23.7%)	7 (9.3%)	0.011^b^	9 (18.4%)	27 (18.2%)	0 (0.0%)	0.157
	Worse	43 (20.8%)	17 (15.9%)	26 (26.0%)		2 (12.5%)	4 (22.2%)	3 (7.7%)	16 (27.1%)	18 (24%)		6 (12.2%)	35 (23.6%)	2 (20.0%)	
	Equal	10 (4.8%)	6 (5.6%)	4 (4.0%)		0 (0.0%)	2 (11.1%)	0 (0.0%)	2 (3.4%)	6 (8.0%)		1 (2.0%)	8 (5.4%)	1 (10.0%)	
	Don’t know	118 (57.0%)	66 (61.7)	52 (52.0%)		8 (50.0%)	9 (50.0%)	30 (76.9%)	27 (45.8%)	44 (58.7%)		33 (67.3%)	78 (52.7%)	7 (70.0%)	
	Total	207 (100%)	107 (100%)	100 (100%)		16 (100%)	18 (100%)	39 (100%)	59 (100%)	75 (100%)		49 (100%)	148 (100%)	10 (100%)	

^a^Also includes GPs still in training.^b^No statistically significant trend observed with the extended Mantel-Haenszel test. *P* values were calculated with χ^2^ tests or likelihood ratio tests. HPV = human papillomavirus. OPC = oropharyngeal cancer.

That HPV-associated OPC patients are generally aged <60 years was correctly recognised by just over half of participants (53.6%). Interestingly, GPs with a self-rated knowledge of ‘good’ were less aware of the younger age of these patients, but no statistically significant trend was observed (*P* = 0.981). Notably, only 17.4% were aware that HPV-associated OPC patients generally have a better prognosis compared with non-HPV-associated OPC patients. Despite the small group size, GPs still in training and/or graduated <2 years ago were more aware of this better prognosis ( 37.5%) compared with their colleagues who graduated >2 years ago: 16.7% for 2–5 years, 15.4% for 5–10 years, 23.7% for 10–20 years, and 9.3% for >20 years after graduation. A trend towards significance was observed (*P* = 0.054). More than half of all GPs did not know about the generally better prognosis of these patients (57.0%) (Table 4).

## Discussion

### Summary

The incidence of HPV-associated OPC is increasing in high income countries, including the Netherlands.^3,6,8,10^ Although these tumours often present with invasive properties and regional lymph node metastases, their prognosis is usually favourable compared with non-HPV-associated tumours.^21^ Early disease recognition by primary care professionals and no delay in the start of treatment are crucial for patient outcomes. The aim of this study was to assess, for the first time, the awareness of the link between HPV and OPC and knowledge of associated patient characteristics in a sample of GPs in the Netherlands. The results show that of the responding GPs: 1) 72.0% were aware of the link between HPV and OPC; 2) 76.3% were aware that HPV-associated OPC rates have increased over the past two decades; and 3) only 35.7% were aware of sex, 53.6% were aware of age, and 17.4% were aware of prognosis of patients with HPV-associated OPC.

### Strengths and limitations

Participants were selected by random sampling of all GPs registered at NIVEL, comprising 85–90% of all GPs in the Netherlands, minimising sampling bias. Furthermore, to minimise response bias, GPs were offered the choice to complete the questionnaire via an online link or on paper. Since the response rate was relatively low, and there is no information on non-responders owing to applied privacy legislation, any (non)response bias that may affect the interpretation of the results of the study cannot be tested. However, it was observed that the percentage of female GPs in the study sample was lower compared with the whole registry of GPs (Supplementary Table S1). Furthermore, participants may have looked at subsequent questions when filling in the paper-format questionnaire, which may have influenced their answers. In the online questionnaire, questions could only be answered in sequence. When comparing the online-format questionnaires with the paper-format questionnaires, however, no difference was observed in awareness of HPV in OPC (73.9% for online versus 71.0% for paper).

### Comparison with existing literature

Previous studies investigating the knowledge on the role of HPV in HNC among medical and dental professionals show varying awareness rates from 26–91%.^26^ The awareness rate of GPs in this study (72%) is comparable to the awareness reported for GPs in the UK (74%) and Poland (80%).^28,31^ The latter study used different outcome variables to assess knowledge of HPV-associated OPC, by asking, 'How important is the impact of HPV on the development of upper respiratory tract pathology?', rather than, 'Have you heard about the link between HPV and OPC before today?' (Table 5). This may induce bias in the interpretation of the actual awareness percentage and could make direct comparison difficult. In contrast, the awareness among GPs in the present study is higher than in Jordan (43.3%), Germany (54%), and Italy (38%)^32–34^ (Table 5). However, these studies were performed >5 years ago and increasing knowledge on HPV and OPC over the years and the introduction of the HPV vaccine might have influenced awareness rates among GPs.

**Table 5.** Overview and results of published studies reporting on awareness of HPV in the development of head and neck cancers among GPs and other healthcare professionals (2014–2018)

Author	Year	Country	Studypopulation	Results
Hertrampf^33^	2014	Germany(Schleswig-Holstein)	33 ENTs,192 GPs,135 IMs,28 DERMs	HPV recognised as a risk factor for oral cancer by 70% of ENTs, 54% of GPs, 51% of IM, and 82% of DERMs
Signorelli^34^	2014	Italy	938 GPs	38% were aware of HPV as a risk factor for oral cancer.
Jackowska^31^	2015	Poland	144 ENTs,192 GPs,68 trainees	Of the GPs, the importance of HPV in the development of OPC was rated as ‘Large’ by 28.6%, as ‘I know the problem’ by 44.8%, as ‘Overrated’ by 6.8%, and as ‘Have not heard about the problem’ by 19.2%.
Hassona^32^	2016	Jordan	165 dentists,165 GPs	43.3% were aware of HPV as a risk factor for oral cancer.No significant difference was found between dentists and GPs
Lechner^28^	2018	UK	384 GPs	73.9% were aware of HPV as a risk factor for OPC

ENT = ear nose and throat. IM = internal medicine. DERM = dermatologist. HPV = human papillomavirus. OPC = oropharyngeal cancer.

The present study showed that the knowledge on HPV-associated OPC patient characteristics and prognosis is limited. The UK study also noticed this knowledge gap, describing that 41.5% of GPs identified HPV-associated OPC as being more common in men, and 58.8% correctly reported the association with younger age.^28^ Interestingly, the results show that GPs in training or recently graduated GPs had greater knowledge of the favourable prognosis. These data suggest that education is necessary to further increase awareness of patient prognosis and demographics of HPV-associated OPC.

Several similar studies among the general population suggest that the awareness of the role of HPV in the development of cervical cancer is relatively high. However, people were shown to be less informed about the role of HPV in OPC.^35–37^ A recent study in the Netherlands showed that 30.6% of 1044 participants had heard of HPV and only 29.2% of these (11.0% of all participants) knew about the association between HPV and OPC.^27^ Importantly, knowledgeable GPs could play an important role in prevention of HPV-associated disease by educating the general public and encouraging the uptake of the HPV vaccine.

### Implications for practice

The results show that the sample of GPs in this study is reasonably aware of HPV as a causative factor for OPC. Nevertheless, more than one-quarter of GPs are still unaware of this link. Specifically, knowledge on less common risk factors and characteristics of patients at risk of HPV-associated OPC should be improved. This knowledge is important as HPV-associated tumours generally present in a relatively young patient population, without typical risk factors, and OPC might therefore be less well recognised in these patients. In terms of educational resources, the authors created a factsheet containing information about HPV and OPC, which was sent to all GPs participating in this study. In addition, further training in the form of regional and national meetings may contribute to better targeted knowledge of these topics, leading to HPV-associated disease prevention, improved disease recognition in the primary care setting, and, ultimately, appropriate referral of patients to secondary care.
